# Bibliometric analysis of global research trends on regulatory T cells in neurological diseases

**DOI:** 10.3389/fneur.2023.1284501

**Published:** 2023-10-12

**Authors:** Qian Gao, Xinmin Li, Yan Li, Junzi Long, Mengyang Pan, Jing Wang, Fangjie Yang, Yasu Zhang

**Affiliations:** ^1^School of Rehabilitation Medicine, Henan University of Chinese Medicine, Zhengzhou, Henan, China; ^2^School of Traditional Chinese Medicine, Henan University of Chinese Medicine, Zhengzhou, Henan, China

**Keywords:** bibliometric analysis, regulatory T cells, neurological diseases, immunomodulation, gut microbiota, cytokines

## Abstract

This bibliometric study aimed to summarize and visualize the current research status, emerging trends, and research hotspots of regulatory T (Treg) cells in neurological diseases. Relevant documents were retrieved from the Web of Science Core Collection. Tableau Public, VOSviewer, and CiteSpace software were used to perform bibliometric analysis and network visualization. A total of 2,739 documents were included, and research on Treg cells in neurological diseases is still in a prolific period. The documents included in the research were sourced from 85 countries/regions, with the majority of them originating from the United States, and 2,811 organizations, with a significant proportion of them coming from Harvard Medical School. Howard E Gendelman was the most prolific author in this research area. Considering the number of documents and citations, impact factors, and JCR partitions, *Frontiers in Immunology* was the most popular journal in this research area. Keywords “multiple sclerosis,” “inflammation,” “regulatory T cells,” “neuroinflammation,” “autoimmunity,” “cytokines,” and “immunomodulation” were identified as high-frequency keywords. Additionally, “gut microbiota” has recently emerged as a new topic of interest. The study of Treg cells in neurological diseases continues to be a hot topic. Immunomodulation, gut microbiota, and cytokines represent the current research hotspots and frontiers in this field. Treg cell-based immunomodulatory approaches have shown immense potential in the treatment of neurological diseases. Modifying gut microbiota or regulating cytokines to boost the numbers and functions of Treg cells represents a promising therapeutic strategy for neurological diseases.

## Introduction

The nervous system serves as the body's command center, and interruptions or impairments of its function can induce neurological diseases, including stroke, spinal cord injury, traumatic brain injury, multiple sclerosis (MS), Alzheimer's disease (AD), and Parkinson's disease (PD) (1). Unfortunately, neurological diseases remain highly prevalent, with a scarcity of effective therapeutic strategies (2, 3). As a result, these disorders pose a considerable socioeconomic burden to society, underscoring the urgent need for continued research and development of effective therapies in the field of neurology. While inflammation may not be the direct cause of neurological diseases, accumulating evidence suggests its involvement in their pathogenesis once these diseases have manifested. A recent research study indicates that disordered innate and adaptive immune responses play a crucial role in the pathological processes of neurological diseases, potentially resulting in autoimmunity, tissue and cellular damage, and subsequent neurological degeneration (4). By addressing the underlying pathological processes that contribute to neurological disease, it is possible to improve the neurological symptoms of individuals affected by these conditions. Therefore, developing effective therapeutic strategies to manage immune-mediated inflammation is crucial in preventing or delaying the onset and progression of neurological diseases.

Regulatory T (Treg) cells are a minor subpopulation of CD4^+^ T cells defined by constitutive expression of IL-2 receptor alpha (CD25) and forkhead box p3 (Foxp3) (5). In 1995, Sakaguchi et al. formally identified Treg cells as a distinct subset of T cells with the CD4^+^CD25^+^ marker (6). This landmark study elucidated the ability of Treg cells to suppress allogeneic responses and revealed that the depletion of Treg cells could lead to enhanced immune responses and the spontaneous development of autoimmune diseases. Treg cells maintain immune homeostasis by suppressing adaptive immune responses and modulating innate immune responses (7). In addition to promoting self-tolerance, Treg cells also mitigate inflammatory conditions to prevent excessive damage to individual tissues.

Early findings suggested that Treg cells could modulate neuroinflammation and attenuate disease progression (6, 8). However, due to the complexity of the immune system and the heterogeneity of neurological diseases, there were discrepancies and conflicting results among individual studies. For example, a study reported that Treg cells, while often beneficial, could act as a double-edged sword in central nervous system injury, attenuating both protective and inflammatory post-injury immune responses and thus either exacerbating or ameliorating neuronal degeneration (9). Several studies identified that Treg cells acted as a negative player in neurological diseases (10, 11). However, numerous studies found that Treg cells play a beneficial role in neurological diseases, and this viewpoint occupies an important position (12–15). In 1996, a study first reported that the oral administration of antigen induces oral tolerance in animal models of experimental autoimmune disease mainly through the induction of Treg cells that actively suppress immune responses by secreting the TGF-β1 cytokine (8). The findings of another study on experimental autoimmune encephalomyelitis indicated that Treg cells play a facilitative role in the remyelination process and exert suppressive effects on neuroinflammatory responses during the chronic stages of MS (12). The expansion of Treg cells has been shown to effectively suppress immune responses and mitigate dopaminergic neurodegeneration in A53T-α-synuclein PD mice (14). Numerous studies have indicated that Treg cells play a beneficial role in delaying the onset and progression of neurological diseases, while dysfunction in Treg cells may lead to the development of autoimmune disease and the progression of neuroinflammation (15). In short, promoting the production and activity of Treg cells represents promising therapeutic strategies for managing immune-mediated inflammation in neurological diseases. Therefore, it is extremely important to understand the current research status and development trends concerning Treg cells in neurological diseases. Gaining such knowledge can help to further explore immunologic mechanisms and therapeutic strategies of neurological diseases and address relevant clinical problems.

Bibliometric analysis can analyze and visualize scientific outputs, research hotspots, and trending topics of a certain field in public literature databases (16). Bibliometric tools, including VOSviewer and CiteSpace, are commonly applied to visualize results of document analysis, which have been widely used in medical fields (17–20). VOSviewer, a free Java-based software, can be used to analyze a large number of document data in an easy-to-interpret way and display it in the form of a map (17). By using CiteSpace, a Java-based software, research results in a certain field can be visualized to help researchers and experts understand the knowledge domain, research frontiers, and development trends (21). Although bibliometric studies on neurological diseases have been conducted, there has yet to be a bibliometric analysis of Treg cells in neurological diseases (22, 23). This study aimed to bridge this knowledge gap by conducting a bibliometric analysis of documents on Treg cells in neurological diseases. Specifically, this analysis identified major contributors and current research status and evaluated future development prospects and research trends in this field.

## Methods

### Data sources and search strategy

All data were downloaded from the Web of Science Core Collection online database, and the search strategy was as follows: TS = (“regulatory T cell^*^” OR “regulatory T-cell^*^” OR “Treg^*^” OR “T-reg^*^”) AND TS = (“neuro^*^”) (23). Subsequently, we limited the document types to articles and review articles, and selected documents written in English. Finally, relevant data were exported in a plain text file with full records and cited references.

### Bibliometric analysis

We analyzed relevant data in the following aspects: the annual number of documents, countries/regions, organizations, authors, journals, keywords, and references. Online platform (http://www.bioinformatics.com.cn) was used to plot the annual document output. Software Tableau Public was applied to draw the geographic distribution of documents. VOSviewer v.1.6.1 and CiteSpace v.6.1.R6 were applied to perform the bibliometric analysis and network visualization, including co-authorship analysis of countries/regions, organizations and authors, co-occurrence analysis of keywords, citation analysis of journals and documents, and co-citation analysis of references.

## Results

### The trend of document outputs

**Figure 1:**
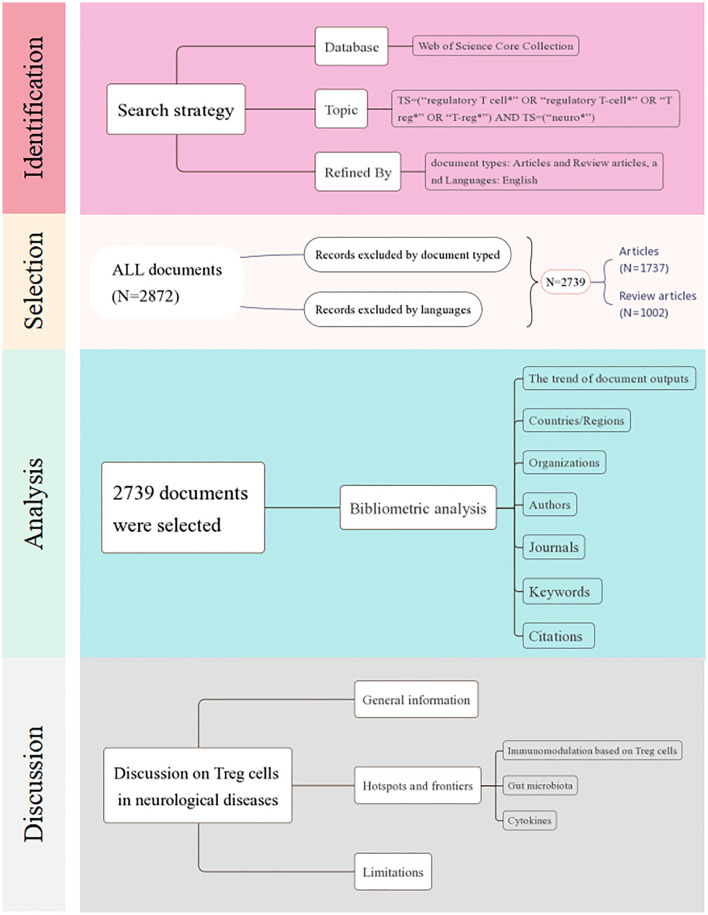
Flowchart of the document-screening process and research framework.

**Figure 2:**
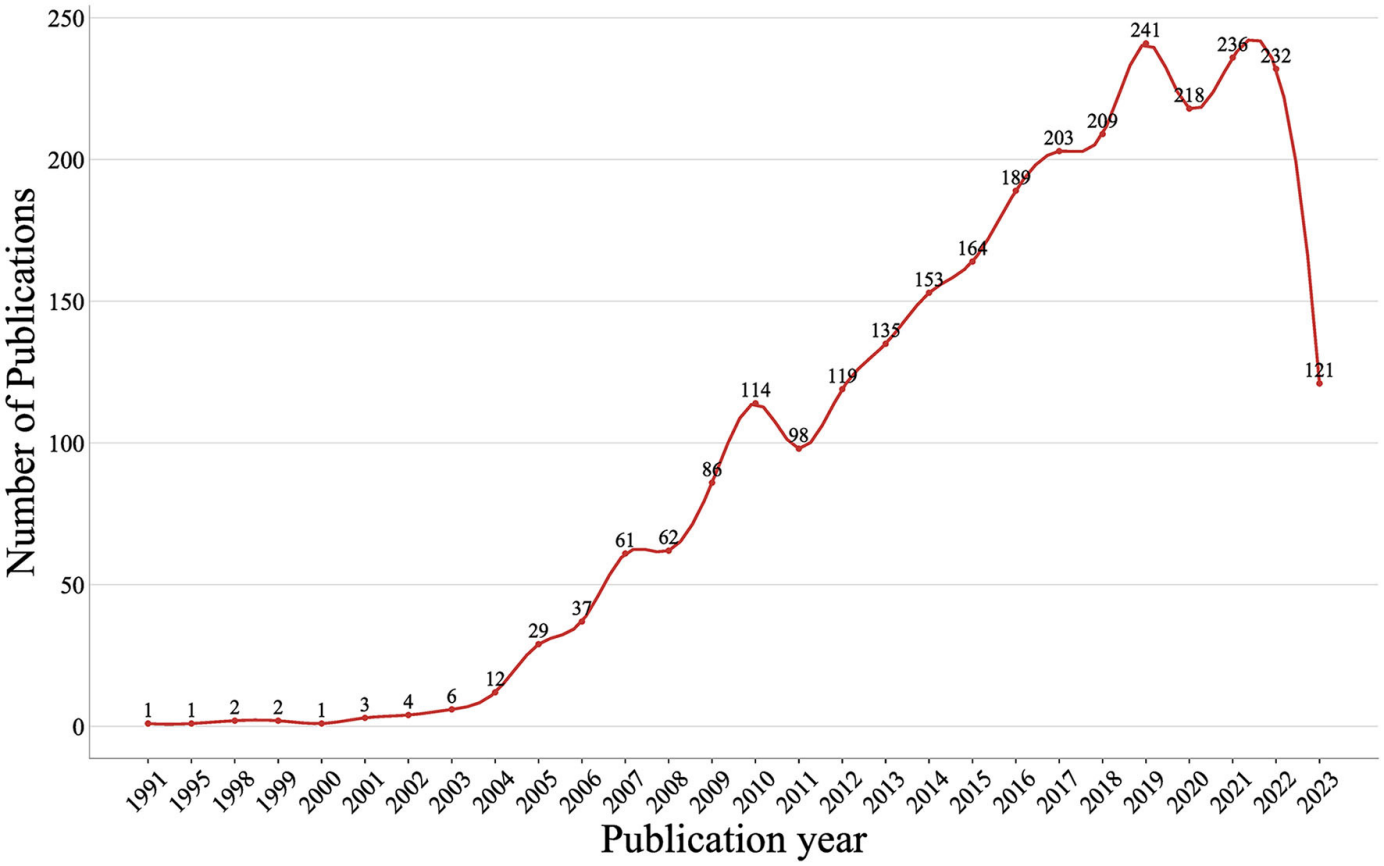
Trends of annual documents related to Treg cells in neurological diseases.

A total of 2,739 documents, including 1,737 articles and 1,002 review articles, were collected from the Web of Science Core Collection on 2 August 2023. The research selection process and research framework are shown in Figure 1. As shown in Figure 2, the research trend can be divided into three stages. During the first stage, spanning from 1991 to 2003 and comprising 20 documents, the output of published research on Treg cells in neurological diseases gradually increased from 1 to 6. This suggests that the field was still in a nascent period, with relatively few studies conducted on the topic at the time. The second stage was from 2004 to 2019, during which the annual output climbed rapidly with a slight fluctuation in 2011. Starting from 2020, the third stage has been a volatile but prolific period in which the annual output of research on Treg cells in neurological diseases has consistently exceeded 210 documents despite fluctuations. Only 7 months of documents were counted in 2023, but the overall trend in published research was stable. It suggested that the role of Treg cells in neurological diseases has attracted extensive attention worldwide since 2004 and remains a continuing hotspot.

### Countries/regions

**Figure 3:**
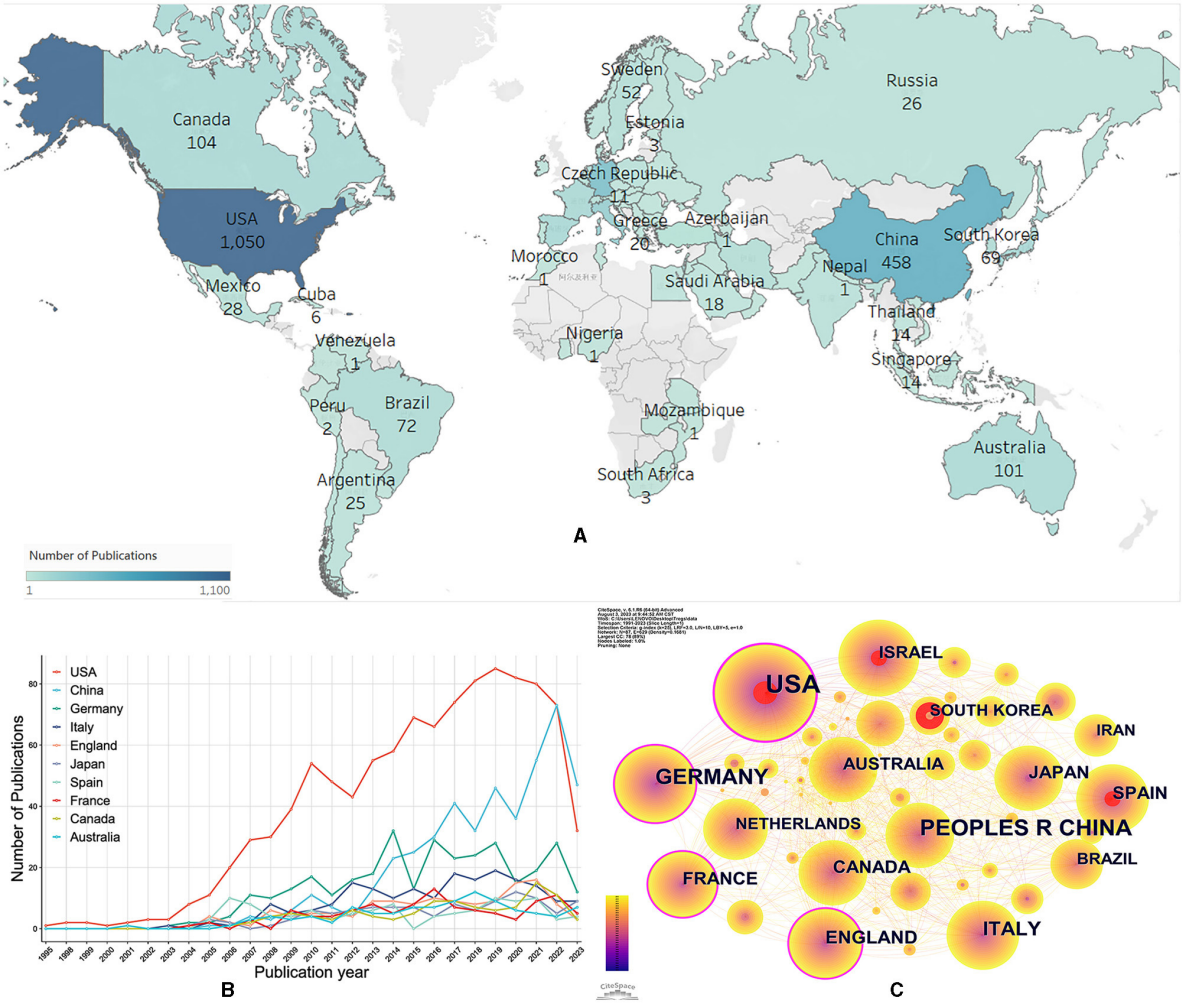
Co-authorship analysis of countries/regions. **(A)** Geographic distribution of documents. The darker the color, the more documents in this country/region. **(B)** Trends of annual documents of the top 10 countries/regions. **(C)** Visualization map of countries/regions collaboration analysis. Each node represents a country/region, and the node size is positively correlated with the number of documents. The connection between nodes represents collaboration. Countries/regions with citation bursts are presented with red nodes, and nodes with purple rings have high centrality values.

A total of 85 countries/regions dabbled in the role of Treg cells in neurological diseases (Figure 3A). The United States, China, and Germany were the top three countries/regions with the most documents on Treg cells in neurological diseases (Table 1). The United States not only had the largest number of documents and citations but also had the highest total link strength and centrality, making it a leading contributor to research on Treg cells in neurological diseases. The annual output of the top 10 countries/regions is shown in Figure 3B. The United States posted a significantly higher annual output than any other country/region until 2021, after which the United States decreased, while China overtook other countries/regions to rank first. In addition, the United States was the earliest country to focus on this research area, while China did not begin to bloom until 2015. The United States, Germany, France, and England had the highest centrality, indicating that they had a strong bridge role in this field (Figure 3C). Some of the documents were completed in cooperation with multiple countries/regions. The United States had collaborated with 57 countries/regions, and Germany had collaborated with 48 countries/regions.

**Table 1:** Top 10 countries/regions with the most documents.

**Rank**	**Country/region**	**Documents**	**Citations**	**Total link strength**	**Centrality**
1	USA	1,050	63,033	554	0.38
2	China	458	12,210	186	0.07
3	Germany	328	20,735	308	0.18
4	Italy	195	10,065	145	0.04
5	England	135	7,240	178	0.1
6	Japan	111	6,519	59	0
7	Spain	108	4,743	100	0.03
8	France	106	4,287	98	0.15
9	Canada	104	6,127	120	0.06
10	Australia	101	5,131	107	0.02

### Organizations

**Figure 4:**
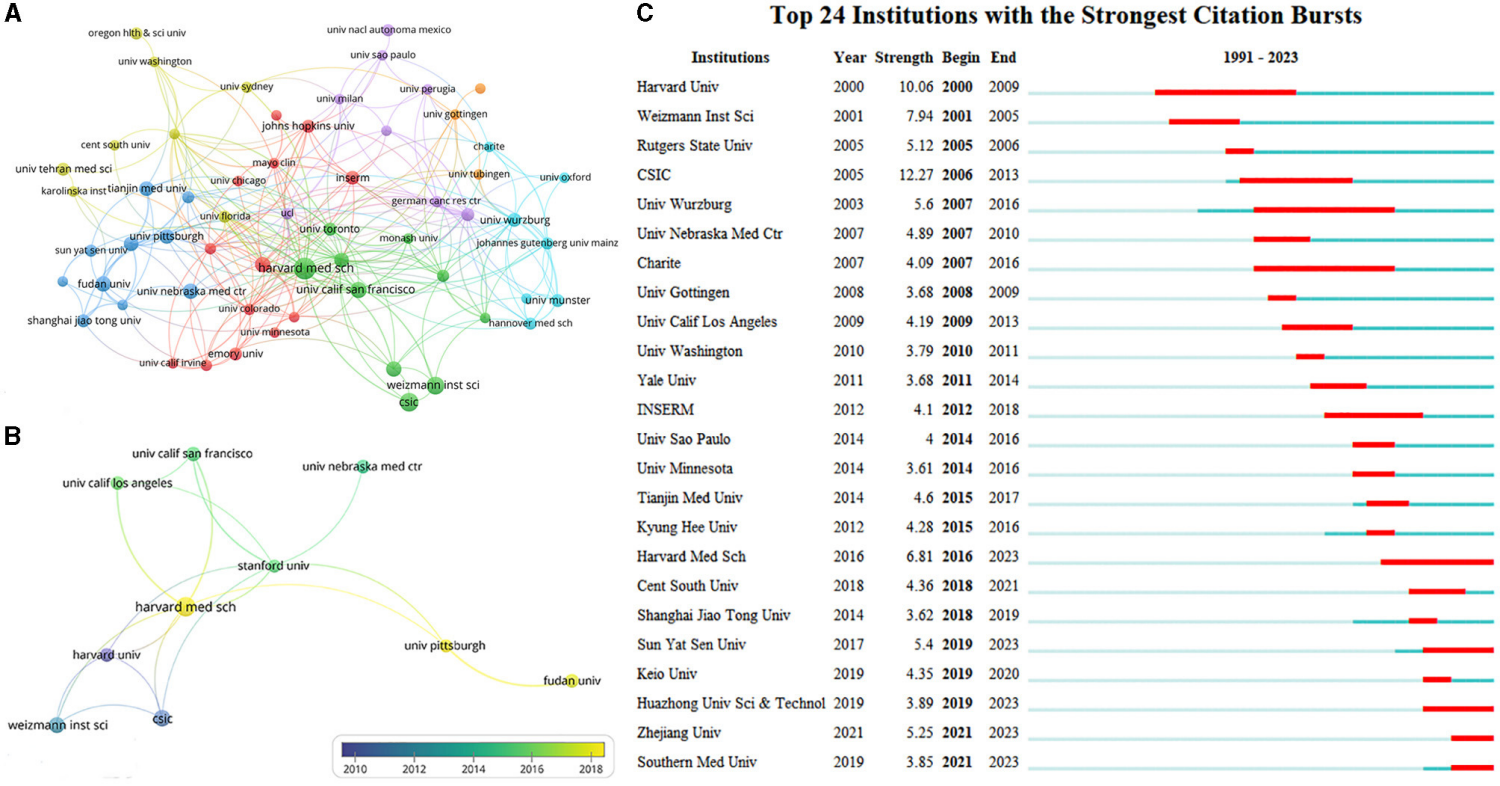
Co-authorship analysis of organizations. **(A)** Visualization map of organizations collaboration analysis. Each node represents an organization, and the node size is positively correlated with the number of documents. The connection between nodes represents collaboration, and the distance and thickness of the connection represent the relative strength of the relationship. **(B)** Visualization map of the top 10 organizations' collaboration. The color means the average published year. **(C)** Top 24 organizations with the strongest citation bursts. Minimum duration: 2.

A total of 2,739 documents were published by 2,811 different organizations, and 61 met the threshold (minimum number of documents of an organization: 15). After excluding disjointed organizations, the remaining 59 organizations were visualized (Figure 4A). The top 10 organizations with the most documents are listed in Table 2, and 7 of the top 10 organizations were affiliated with the United States. Harvard Medical School ranked first in terms of the number of documents and citations, total link strength, and centrality, indicating that it was the most prolific organization and had the most cooperation with other organizations. In addition, the University of California system, including the University of California San Francisco (UCSF) and the University of California Los Angeles (UCLA), was another important organization in this research area. As shown in Figure 4B, Harvard Medical School, University of Pittsburgh, and Fudan University (nodes with yellow color) were the most recent organizations to publish more documents. The top three organizations with the strongest citation bursts were Consejo Superior de Investigaciones Cientificas (CSIC) from 2006 to 2013, Harvard University from 2000 to 2009, and Weizmann Institute of Science from 2001 to 2005 (Figure 4C). The citation bursts in many organizations have continued until 2023, suggesting that Treg cells in neurological diseases remain the hotspot for future research by many organizations.

**Table 2:** Top 10 organizations with the most documents.

**Rank**	**Organization**	**Country/Region**	**Documents**	**Citations**	**Total link strength**	**Centrality**
1	Harvard Medical School	USA	64	4,019	43	0.17
2	Consejo Superior de Investigaciones Cientificas (CSIC)	Spain	47	1,814	5	0.04
3	Weizmann Institute of Science	Israel	45	2,586	6	0.03
4	University of California San Francisco	USA	36	3,426	23	0.05
5	Fudan University	China	35	1,482	16	0.04
6	University of Nebraska Medical Center	USA	35	1,801	3	0.01
7	University of California Los Angeles	USA	34	1,459	26	0.03
8	Harvard University	USA	33	3,543	10	0.09
9	Stanford University	USA	31	2,717	39	0.13
10	University of Pittsburgh	USA	31	2,394	21	0.09

### Authors

**Figure 5:**
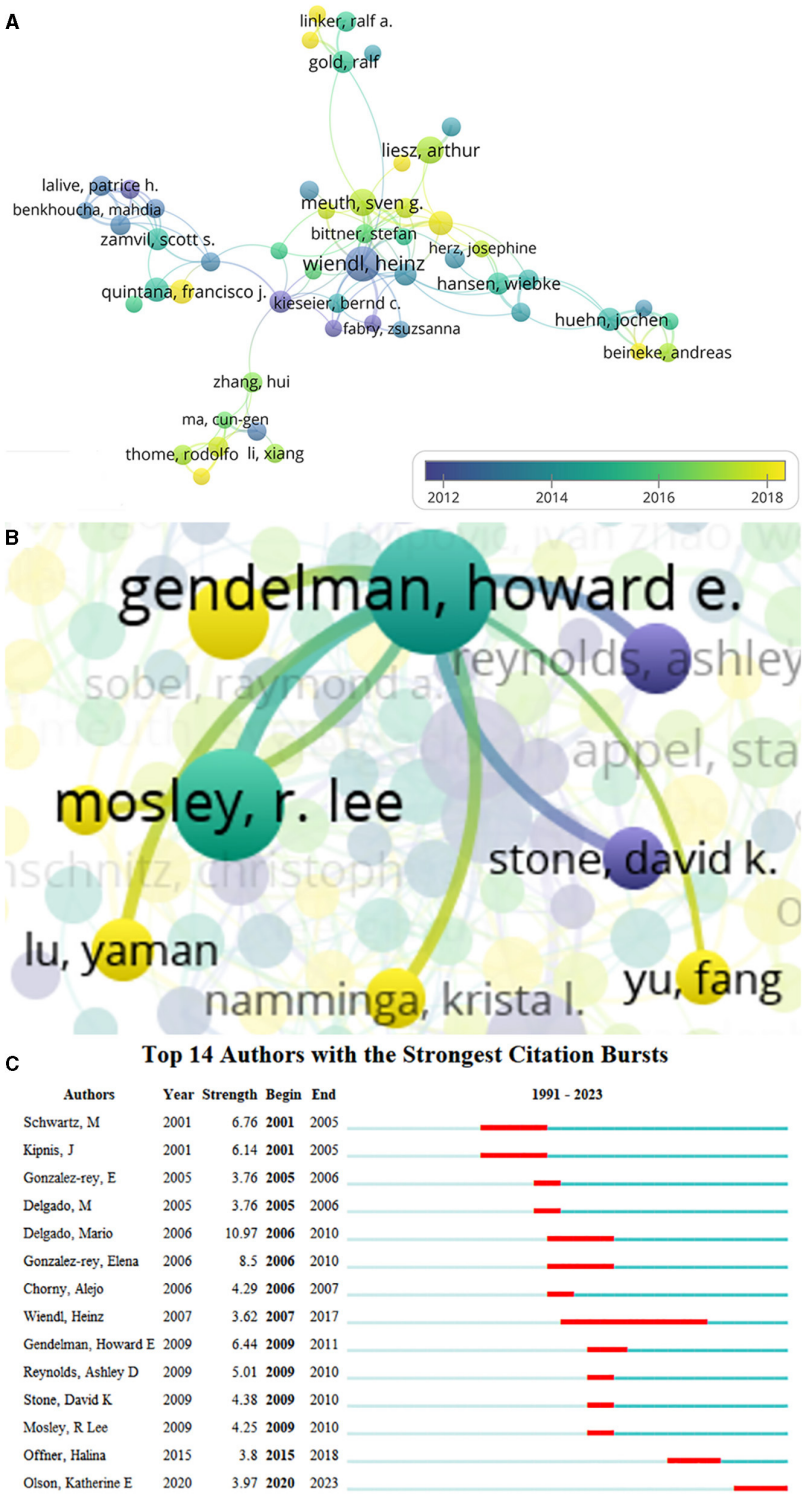
Co-authorship analysis of authors. **(A)** Visualization map of authors collaboration analysis. Each node represents an author, and the node size is positively correlated with the number of documents. The connection between nodes represents collaboration, and the distance and thickness of the connection represent the relative strength of the relationship. The color means the average published year. **(B)** Cooperative network of Gendelman HE. **(C)** Top 14 authors with the strongest citation bursts. Minimum duration: 2.

A total of 13,859 authors were involved in Treg cells in neurological diseases, and 185 met the threshold (minimum number of documents of an author: 5). The largest set of connected items consisted of 51 authors (Figure 5A). The top 15 core authors in this field are listed in Table 3, of which 6 authors came from the United States. The top 15 authors published 271 documents, accounting for 9.89% of the total number. Howard E Gendelman and R Lee Mosley were the top authors with the largest number of documents and citations. Both are affiliated with the University of Nebraska Medical Center in the United States, which ranked sixth in terms of the number of documents about the role of Treg cells in neurological diseases. As shown in the visualization map of authors (Figure 5A), Heinz Wiendl was the center of authors' co-authorship relations and had the longest citation bursts. However, Howard E Gendelman was not involved in the largest connected cooperative network. A total of eight authors cooperated with Howard E Gendelman, and they were all affiliated with the University of Nebraska Medical Center (Figure 5B). Several emerging scholars (nodes with yellow color) have also begun to dabble in this field, suggesting that Treg cells in neurological diseases are still a hotspot for future research. The top three authors with the strongest citation bursts were Marina Delgado from 2006 to 2010, Michal Schwartz from 2001 to 2005, and Howard E Gendelman from 2009 to 2011, indicating that they were leaders in this field in a certain period (Figure 5C).

**Table 3:** Top 15 authors with the most documents.

**Rank**	**Author**	**Organization**	**Country/Region**	**Documents**	**Citations**	**Total link strength**
1	Gendelman HE	University of Nebraska Medical Center	USA	34	1,935	80
2	Mosley RL	University of Nebraska Medical Center	USA	27	1,655	76
3	Delgado M	Consejo Superior de Investigaciones Cientificas (CSIC)	Spain	26	859	48
4	Gonzalez-rey E	Consejo Superior de Investigaciones Cientificas (CSIC)	Spain	24	806	51
5	Bae H	Kyung Hee University	Korea	20	529	33
6	Wiendl, H	University of Münster	Germany	20	1,217	38
7	Offner H	VA Portland Health Care System, Oregon Health & Science University	USA	17	687	23
8	Schwartz M	The Weizmann Institute of Science	Israel	17	1,242	4
9	Olson KE	University of Nebraska Medical Center	USA	14	402	44
10	Appel SH	Houston Methodist Research Institute	USA	12	1,078	25
11	Fragoso G	Univ Nacl Autonoma Mexico	Mexico	12	173	40
12	Hu X	University of Pittsburgh School of Medicine	USA	12	749	27
13	Kipnis J	The Weizmann Institute of Science	Israel	12	569	2
14	Liesz A	Heidelberg University	Germany	12	1,251	10
15	Meuth SG	Heinrich-Heine University of Düsseldorf	Germany	12	606	25

### Journals

A total of 859 journals published 2,739 documents concerning Treg cells in neurological diseases. The top 11 journals are listed in Table 4, they published 618 documents, accounting for approximately 22.56% of the total. *Frontiers in Immunology* with 134 documents, *Journal of Immunology* with 90 documents, and *Journal of Neuroinflammation* with 75 documents were the most prolific journals. Impact factors of the top 11 journals ranged from 3.3 to 15.1, of which *Brain Behavior and Immunity* was the highest, and *Journal of Neuroimmunology* was the lowest. Of the top 11 journals, 6 journals belonged to Q1, 4 journals belonged to Q2, and the remaining 1 journal belonged to Q3. Notably, the *Journal of Experimental Medicine*, with the most citations (4,352 times), was not among the top 11 journals. The document “HIF1 alpha-dependent glycolytic pathway orchestrates a metabolic checkpoint for the differentiation of T(H)17 and T-reg cells” published in this journal in July 2011 was cited 1215 times, which ranked second in terms of the number of citations. Although the number of documents published in the *Journal of Experimental Medicine* was relatively small, the quality of documents was relatively high, which had conspicuously pushed forward the progress in this field. In short, both the number and the quality of documents need to be considered in the evaluation of prolific journals. Considering the number of documents and citations, impact factors, and JCR partitions, *Frontiers in Immunology* was the most popular journal in this research area.

**Table 4:** Top 11 journals with the most documents.

**Rank**	**Journal**	**Documents**	**Citations**	**Total link strength**	**Impact factor (2022)**	**JCR partition**
1	Frontiers in Immunology	134	4,351	603	7.3	Q1
2	Journal of Immunology	90	4,323	463	4.4	Q2
3	Journal of Neuroinflammation	75	3,057	405	9.3	Q1
4	Journal of Neuroimmunology	72	2,117	335	3.3	Q3
5	International Journal of Molecular Sciences	47	1,273	212	5.6	Q1
6	PLoS One	46	1,925	199	3.7	Q2
7	Brain Behavior and Immunity	38	1,440	188	15.1	Q1
8	Scientific Reports	33	955	116	4.6	Q2
9	Journal of Neuroimmune Pharmacology	29	1,144	161	6.2	Q1
10	Immunology	27	1,365	148	6.4	Q2
11	Proceedings of the National Academy of Sciences of the United States of America	27	3,238	299	11.1	Q1

### Keywords

**Figure 6:**
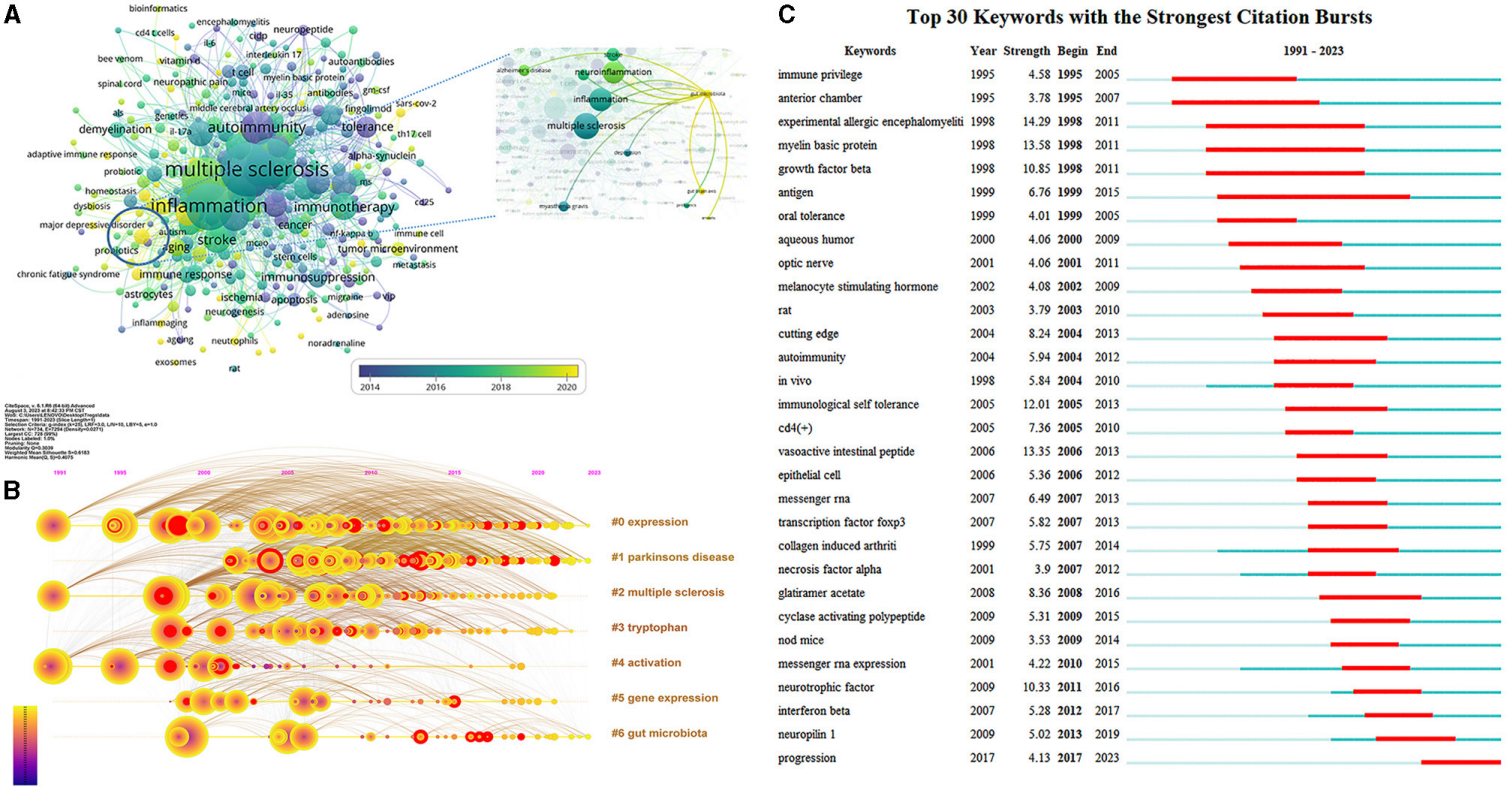
Co-occurrence analysis of keywords. **(A)** Visualization map of author keywords analysis. Each node represents an author keyword, and the node size is positively correlated with the number of documents containing the author keyword. The connection between nodes represents co-occurrence. The color means the average published year. **(B)** Timeline view of keywords clustering analysis. The different colored horizontal lines on the right represent the clusters formed by the keywords, nodes on the horizontal lines represent keywords, and the position of nodes on the horizontal lines represents the year in which the document containing the keywords first appeared. **(C)** Top 30 keywords with the strongest citation bursts. Minimum duration: 6.

A total of 4,868 author keywords were involved in 2,558 documents, and 354 met the threshold (minimum number of documents of a keyword: 5). The overlay visualization map showed the co-occurrence relations of keywords (Figure 6A), in which “multiple sclerosis,” “inflammation,” “regulatory T cells,” “neuroinflammation,” “autoimmunity,” “microglia,” “cytokines,” “experimental autoimmune encephalomyelitis,” “immunotherapy,” and “immunomodulation” were identified as high-frequency keywords. Moreover, these keywords were mostly associated with neuroprotection, neuroimmunology, and immunoregulation in 2014; interconnected with myasthenia gravis, MS, and neurodegeneration in 2016; related to PD, AD, and spinal cord injury in 2018; and currently linked to ischemic stroke, gut microbiota, and the gut–brain axis. Recently, the role of gut microbiota in neurological diseases has gained significant attention, with substantial evidence linking it to neuroinflammation.

As shown in Figure 6B, the timeline view of keywords clustering analysis was displayed to show the basic knowledge structure and the evolution over time of Treg cells in neurological diseases. The modularity Q was 0.4075, indicating that the network structure was consequential, and the mean silhouette S was 0.6183, implying that clustering was credible. Keywords with close relationship were automatically grouped into a cluster, which was named by the keyword with the largest log-likelihood rate. Cluster “#0 expression,” “#2 multiple sclerosis,” and “#4 activation” appeared the earliest, and cluster “#1 Parkinsons disease” appeared the latest. Cluster “#0 expression,” “#1 Parkinson's disease,” and “#6 gut microbiota” related studies were available in 2023, which may become frontiers of Treg cells in neurological diseases in future, while cluster “#2 multiple sclerosis,” “#3 tryptophan,” “#4 activation,” and “#5 gene expression” gradually decreased or even disappeared.

The top 30 keywords with the strongest citation bursts are shown in Figure 6C, which were considered consequential milestones for the science mapping research. “Immune privilege” and “anterior chamber” were important contents of the earliest research, suggesting that the immune privilege of the anterior chamber was an early research hotspot and had occupied a major position in this field. Keywords “antigen” had the longest 16 years of duration burst. In addition, “experimental allergic encephalomyelitis” had the highest burst strength from 1998 to 2011, which implied that scholars can never ignore its equally important existence when conducting research in this field, followed by “myelin basic protein” and “vasoactive intestinal peptide”.

### Citations

The top 10 documents with the most citations are listed in Table 5, and the range of citations was from 597 to 2,058. The top three documents with the most citations were documents written by Scheller J in 2011 (24), Shi LZ in 2011 (25), and Setoguchi R in 2005 (26), whereby all introduced the role of cytokines in autoimmune neurological diseases. The document written by Lee YK in 2011 (27), pointed out that gut microbiota impacts the balance between pro-and anti-inflammatory immune responses during experimental autoimmune encephalomyelitis. The document written by Wang MN in 2017 (28) focused on the role of tumor microenvironment in tumorigenesis of glioma, glioblastoma, and other cancers. The documents written by Lakhan SE in 2009 (29), Karussis D in 2010 (30), and Haroon E in 2012 (32) introduced the immunologic mechanisms underlying several therapeutic approaches for neurological diseases, such as ischemic stroke, MS, amyotrophic lateral sclerosis, and depression.

**Table 5:** Top 10 documents with the most citations.

**Rank**	**Title**	**References**	**Journal**	**Citations**
1	The pro- and anti-inflammatory properties of the cytokine interleukin-6	Scheller et al. (24)	Biochimica Et Biophysica Acta-Molecular Cell Research	2,058
2	HIF1 alpha-dependent glycolytic pathway orchestrates a metabolic checkpoint for the differentiation of T(H)17 and T-reg cells	Shi et al. (25)	Journal of Experimental Medicine	1,215
3	Homeostatic maintenance of natural Foxp3(+) CD25(+) CD4(+) regulatory T cells by interleukin (IL)-2 and induction of autoimmune disease by IL-2 neutralization	Setoguchi et al. (26)	Journal of Experimental Medicine	941
4	Proinflammatory T-cell responses to gut microbiota promote experimental autoimmune encephalomyelitis	Lee et al. (27)	Proceedings of the National Academy of Sciences of the United States of America	910
5	Role of tumor microenvironment in tumorigenesis	Wang et al. (28)	Journal of Cancer	798
6	Inflammatory mechanisms in ischemic stroke: therapeutic approaches	Lakhan et al. (29)	Journal of Translational Medicine	705
7	Safety and Immunologic Effects of Mesenchymal Stem Cell Transplantation in Patients with Multiple Sclerosis and Amyotrophic Lateral Sclerosis	Karussis et al. (30)	Archives of Neurology	679
8	The Immunomodulatory and Anti-Inflammatory Role of Polyphenols	Yahfoufi et al. (31)	Nutrients	676
9	Psychoneuroimmunology Meets Neuropsychopharmacology: translational Implications of the Impact of Inflammation on Behavior	Haroon et al. (32)	Neuropsychopharmacology	626
10	Effects of stress on immune function: the good, the bad, and the beautiful	Dhabhar (33)	Immunologic Research	597

**Figure 7:**
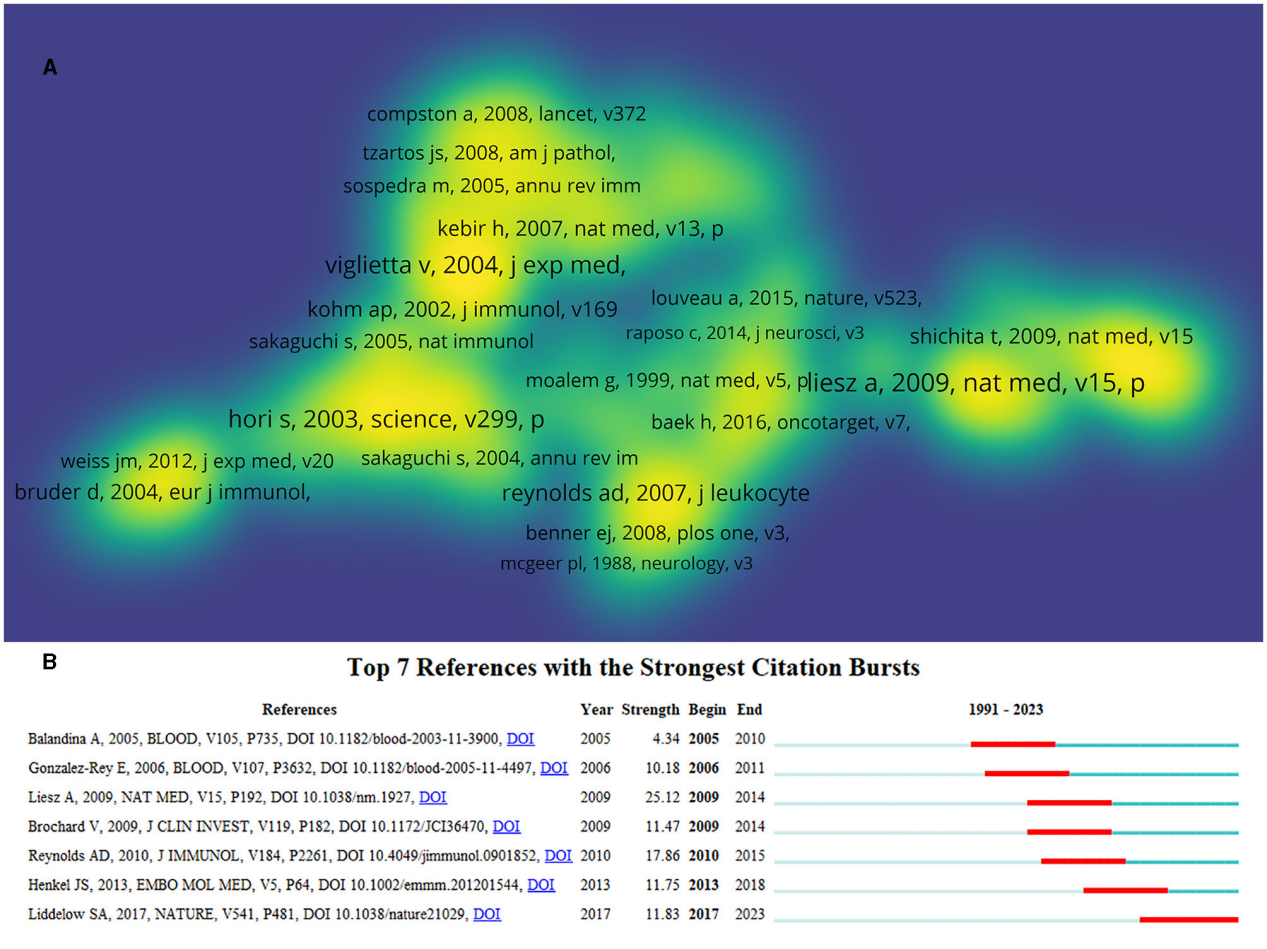
Co-citation analysis of cited references. **(A)** The density map of cited references based on citations. The opacity of yellow is positively related to citations. **(B)** Top 7 references with the strongest citation bursts. Minimum duration: 6.

The co-citation analysis of cited references was performed by VOSviewer. A total of 162,113 cited references were involved in 2,739 documents, and 131 met the threshold (minimum number of citations of a cited reference: 40). The density visualization map of cited references based on citations is shown in Figure 7A, and the top 10 cited references with the most citations are shown in Table 6. The reference with the most citations was an article written by Liesz A in 2009, which indicated that research in this article may be a research hotspot, followed by references written by Viglietta V in 2004 and Hori S in 2003. Among the top 10 cited references, five references (34, 35, 39, 42, 43) focused on the neuroprotective role of Treg cells in neurological diseases, including stroke, MS, and PD. In total, five references (34, 36, 37, 40, 41) highlighted the important role of cytokines, including Foxp3, IL-10, IL-2, and TGF-β, in the generation, development, and function of Treg cells, suggesting that cytokines have always been the research focus in this field.

**Table 6:** Top 10 references with the most citations.

**Rank**	**Title**	**References**	**Journal**	**Citations**
1	Regulatory T cells are key cerebroprotective immunomodulators in acute experimental stroke	Liesz et al. (34)	Nature Medicine	250
2	Loss of functional suppression by CD4(+)CD25(+) regulatory T cells in patients with multiple sclerosis	Viglietta et al. (35)	Journal of Experimental Medicine	199
3	Control of regulatory T cell development by the transcription factor Foxp3	Hori et al. (36)	Science	174
4	Foxp3 programs the development and function of CD4(+)CD25(+) regulatory T cells	Fontenot et al. (37)	Nature Immunology	155
5	Regulatory T cells and immune tolerance	Sakaguchi et al. (38)	Cell	143
6	Neuroprotective activities of CD4+CD25+ regulatory T cells in an animal model of Parkinson's disease	Reynolds et al. (39)	Journal of Leukocyte Biology	138
7	Reciprocal developmental pathways for the generation of pathogenic effector T(H)17 and regulatory T cells	Bettelli et al. (40)	Nature	125
8	Immunologic self-tolerance maintained by activated T cells expressing IL-2 receptor alpha-chains (CD25). Breakdown of a single mechanism of self-tolerance causes various autoimmune diseases	Sakaguchi et al. (41)	Journal of Immunology	120
9	Regulatory T Cells Attenuate Th17 Cell-Mediated Nigrostriatal Dopaminergic Neurodegeneration in a Model of Parkinson's disease	Reynolds et al. (42)	Journal of Immunology	111
10	The immunology of stroke: from mechanisms to translation	Iadecola et al. (43)	Nature Medicine	103

Reference burst detection can help find the most influential cited references and discover research frontiers and trends. In total, seven references with the strongest citation bursts were obtained (Figure 7B). The reference written by Liesz A in 2009 had the highest burst and highest number of citations, indicating that the research discussed in this article is authoritative and has been a hotspot in this field. Judging from the past 6 years, a reference published by Liddelow SA in 2017 (44) has become the latest research frontier so far and may continue in the next decade. This reference titled “Neurotoxic reactive astrocytes are induced by activated microglia,” suggested that inflammatory cells contribute to the death of neurons in AD, PD, amyotrophic lateral sclerosis, and MS and provided opportunities for the development of cell-based immunotherapies for these diseases.

## Discussion

### General information

Annual documents on Treg cells in neurological diseases showed an overall upward trend, suggesting that this research field remains an active hotspot. Among 85 countries/regions publishing documents on this topic, the United States was the largest contributor, with double the number of documents and citations compared to China and far ahead of other countries/regions. Additionally, among the top 10 most productive organizations, seven were based in the United States, and among the top 15 most prolific authors, six were also from the United States, underscoring their substantial contributions to this research field. However, China has emerged as a potential contributor in this field, with its annual output overtaking other countries/regions and ranking first in 2022. Harvard Medical School was identified as the most important organization and a major driver of research on the role of Treg cells in neurological diseases. Nearly 25% of relevant research results were published in the top 11 journals, demonstrating their high quality and authoritative role as communication platforms for research related to Treg cells in neurological diseases. Notably, *Frontier in Immunology* was the most popular journal, playing an active role in promoting the development of Treg cells in neurological diseases. Howard E Gendelman, currently affiliated with the University of Nebraska Medical Center, has published the most documents on Treg cells in neurological diseases. These documents primarily focused on neuroimmunity, neuromodulatory, immunomodulation, and neuroprotection. Among these documents, the document “Regulatory T cells attenuate Th17 cell-mediated nigrostriatal dopaminergic neurodegeneration in a model of Parkinson's disease” has achieved the most citations. This study highlighted the potential of Treg cells in regulating neurodestructive immunity and laid the foundation for immunization strategies for PD (42).

There has been a remarkable historical progression in the field of Treg cells in neurological diseases. Initially, research focused on characterizing Treg cells and their role in maintaining immune homeostasis (6, 8). Over time, studies began to explore their involvement in neurological diseases, such as MS, AD, and PD (8, 12–14). Furthermore, this research area has undergone a significant transformation, shifting from the concept of neuroprotective autoimmunity to neuroprotection through neuroimmune transformation. A previous research study focused on the detrimental role of Th17/Th1 cells and the protective role of Treg cells, leading to strategies targeting Th17/Th1 suppression and Treg activation (45, 46). However, our deepening understanding emphasizes the critical balance between Th17/Th1 and Treg cells for neuroprotection. Under specific conditions, these cell subpopulations can convert into each other, playing a pivotal role in balancing immune effects and suppression (47, 48). Correcting Th17/Treg cell imbalance is now a novel approach for disease prevention and treatment (49, 50). As the field evolved, researchers made significant contributions by refining experimental models, developing more precise methodologies to identify and track Treg cells in the central nervous system, and investigating their specific mechanisms of action. However, this research area is still in the development stage and has enormous development potential. To gradually advance the field of Treg cells in neurological diseases and form a consensus, independent investigators, organizations, and countries should prioritize standardizing experimental conditions, including cell sources, assay protocols, and animal models to ensure the replicability and extensibility of their studies. Second, while striving for progress, researchers should actively incorporate diverse perspectives, such as conducting systematic reviews, collaboratively evaluating existing literature, and using data-driven approaches to resolve discrepancies. Importantly, researchers can explore whether Treg cells from different sites and sources exhibit distinct roles or whether there are different phenotypes of Treg cells with specialized functions (51–53).

### Hotspots and frontiers

Keywords are powerful tools for understanding the theme and research focus of scientific documents, and they can help identify hotspots and trends of Treg cells in neurological diseases. The most cited documents often signify important research directions and breakthroughs in the field. Co-cited references reflect the historical development and roots of the field, while references with citation bursts reveal the emerging hotspots within it. By combining keyword and citation analyses, we have identified the following aspects as current research hotspots and trends of Treg cells in neurological diseases:

#### Immunomodulation based on Treg cells

Given their immunosuppressive properties, Treg cells are considered excellent candidates for immunomodulation. Treg cell-based therapeutic strategies have been actively developing in transplantation and autoimmune diseases (46). The absence of Treg cells in the lymphoid aggregates of MS patients' brains indicates that the reduction of Treg cells may play a role in the progression of the disease (54). Thus, therapies based on Treg cells have the potential to ameliorate MS. A phase I clinical trial evaluating the adoptive transfer of Treg cells into patients with relapsing-remitting MS found it to be safe and well-tolerated, without adverse events (55). Nonetheless, additional research is necessary to assess the efficacy and safety of Treg cell-based therapeutic strategies for patients with MS, given the limited knowledge about how Treg cells influence immune homeostasis and inflammation resolution. The early depletion of Treg cells by anti-CD25 antibody hastened cognitive deficits in APP/PS1 mice and reduced microglial recruitment to amyloid deposits (56). The adaptive transfer of *ex vivo* expanded human Treg cells to immunodeficient 5xFAD-Rag2KO mice resulted in the suppression of neuroinflammation and significant alleviation of amyloid pathogenesis (13). In disagreement, a study suggested that transient Treg cell depletion was followed by amyloid-β plaque clearance, mitigation of the neuroinflammatory response, and reversal of cognitive decline in the AD mouse model (10). Although there are disagreements regarding the role of Treg cells in the progression of AD, it is undeniable that Treg cell modulation is a new treatment option. PD is a neurodegenerative disorder characterized by neuroinflammation that may be caused by an imbalance between Treg cells and Th17 cells. Treg cells have been shown to attenuate Th17 cell-mediated death of nigrostriatal dopaminergic neurons (57). An *in vitro* study revealed that human adipose tissue-derived mesenchymal stem cells could inhibit the differentiation of CD4^+^ T cells isolated from patients with PD into Th17 cells. This inhibitory effect was mainly mediated by an increase in Treg cells and secretion of IL-10, indicating that Treg cells play an anti-inflammatory and neuroprotection role in PD (49). Immunomodulation through Treg cell expansion was found to be an effective treatment for PD mice in a recent study, providing evidence that immunotherapy may offer a disease-modifying option for patients with PD (14). While a study demonstrated that depleting Foxp3^+^ Treg cells in transgenic DEREG mice significantly reduced lesion volume and improved neurological function during the early phase of middle cerebral artery occlusion (11), many studies have shown that an increase in Treg cells could potentially improve long-term stroke recovery (46, 58, 59). Researchers found that Treg cell-derived osteopontin contributed to a tissue-reparative microglial response. This response led to improved oligodendrocyte regeneration and remyelination during the chronic stages of stroke (58). The use of a CD28 superagonist to expand and amplify Treg cells attenuated the inflammatory response, reduced infarct volume, and improved outcomes in experimental stroke (59).

Recently, engineered Treg cells have been used for adoptive immunotherapy. First, human Treg cells are isolated from human peripheral blood, umbilical cord blood, or thymus. These Treg cells are then cultured *in vitro* to generate polyclonal Treg cells or antigen-specific Treg cells. Finally, qualified Treg cells are infused into patients to treat related diseases (60). Therefore, immunomodulatory strategies based on Treg cells are novel and promising therapies for neurological diseases and deserve continued research by scholars.

#### Gut microbiota

Substantial evidence has indicated that the gut–brain axis likely plays a crucial role in neurological diseases, with an altered gut microbiota potentially having significant implications on immune responses in both the gut and distal effector immune sites such as the central nervous system (61). A study involving experimental autoimmune encephalomyelitis mice found that the gut microbiota greatly influenced the balance between pro- and anti-inflammatory immune responses. This discovery suggested that modulating gut microbiota could provide new targets for treating extraintestinal inflammatory diseases such as MS (27). Specific metabolites of gut microbiota, such as the tryptophan metabolite FICZ [6-formylindolo (3-2b) carbazole], are associated with the production of pro-inflammatory cytokines and the generation of Th17 cells. Conversely, commensal bacteria and their metabolites, including *Lactobacilli* and *Bacillus*-derived poly-gamma-glutamic acid (gamma-PGA), can stimulate Treg cell generation to promote immune suppression. Therefore, the immunomodulatory effects of gut microbiota may be mediated primarily *via* the Th17/Treg axis (62). Exposure to MS microbiota or MS-associated *Acinetobacter calcoaceticus* extract was shown to alter lymphocyte differentiation in healthy individuals, resulting in an increase in Th1 cells and a decrease in CD25^+^Foxp3^+^ Treg cells, while exposure to the *Parabacteroides distasonis* extract increased Treg cell differentiation (63). Patients with MS display a reduction in commensal microbiota levels compared to healthy individuals, and therapies targeting the microbiota have been demonstrated to increase the microbiota and improve MS by decreasing Th1- and Th17-cell levels and increasing Treg cell levels (64). Myasthenia gravis is an autoantibody-mediated neurological disease, and Th17/Treg imbalance contributes to the pathogenesis of myasthenia gravis. Studies have reported that correcting Th17/Treg imbalances may be a novel therapeutic approach to myasthenia gravis by modifying the gut microbiota (50). Butyrate promotes the expression of Foxp3 and differentiates naive T cells into Treg cells by inhibiting histone deacetylase. Therefore, the reduction of short-chain fatty acid-producing bacteria in patients with PD reduces the number of Treg cells, thereby exacerbating the neuroinflammatory response (65). Patients with neurological diseases often exhibit gut microbial dysbiosis and altered microbial metabolites, highlighting the potential of microbial components or commensal bacteria as immunomodulatory agents to correct Th17/Treg imbalances and then treat neurological diseases (50). Therefore, developing therapeutic interventions targeting the gut microbiome could represent a promising strategy for managing neurological diseases.

#### Cytokines

Cytokines are under active investigation as immune modulators to boost the numbers and functions of Treg cells in neurological diseases. The development and function of CD4^+^CD25^+^ Treg cells are regulated by Foxp3, while peripheral CD4^+^CD25^−^ T cells can acquire suppressor function through ectopic Foxp3 expression. This discovery opens up a new way for cell-based therapies for autoimmunity (37). IL-2 is an essential factor for the development, survival, and function of Foxp3^+^ natural Treg cells, playing a critical role in maintaining Treg cell homeostasis (26, 38). Studies have revealed that low-dose IL-2 therapy can selectively promote the persistence and survival of Treg cells while limiting effects on other T-cell subsets. The therapeutic efficacy of this approach has been demonstrated in both animal models and clinical trials, highlighting its potential as a promising treatment option (66, 67). The aberrant TGF-β signaling observed in individuals with MS is strongly associated with Treg cell dysfunction (68). Consequently, targeting and modulating TGF-β signaling may hold promise for addressing this defect and potentially alleviating the symptoms of MS. IL-6 plays a pivotal role in regulating the balance between Th17 and Treg cells. Specifically, IL-6 supports the differentiation of Th17 cells from naive T cells together with TGF-β and inhibits TGF-β-induced Treg differentiation (69). Tocilizumab, an anti-IL-6 receptor monoclonal antibody, has been approved for treating inflammatory diseases (24). Therefore, the utilization of cytokines as immune modulators to regulate the differentiation and function of Treg cells represents a significant therapeutic approach in the treatment of neurological diseases. Furthermore, relevant immunomodulatory agents have transformed recent clinical practice to prevent and reverse the pathology of neurological diseases. However, a delivery system that can cross the blood–brain barrier to carry immunomodulatory agents is still the direction of scholars' unremitting exploration.

### Limitations

This study is the first bibliometric analysis to systematically analyze documents related to Treg cells in neurological diseases. Nevertheless, there are still some deficiencies here. First, only English language articles and reviews published in the Web of Science Core Collection were collected, which may lead to language and publication bias. As bibliometric analysis is closely linked to timeliness, it is essential to continuously update the results and trends of research on Treg cells in neurological diseases to keep pace with ongoing scientific exploration. This will enable a more comprehensive understanding of the topic as well as provide more precise predictions of future trends. Finally, this review discusses from the perspective of the neuroprotective role of Treg cells, however, varying perspectives exist in certain studies. Therefore, before reaching a consensus, it is important to consider multiple aspects when targeting Treg cells for the treatment of neurological diseases and exercise caution in their use. However, given the large enough number of documents in this analysis, we believe that this study provides an instructive perspective for the research of Treg cells in neurological diseases and guides future research in this field.

## Conclusion

Through VOSviewer, CiteSpace, and Tableau Public software, we have carried out a bibliometric analysis on Treg cells in neurological diseases. The study of Treg cells in neurological diseases continues to be a hot topic. The United States was the largest contributor among 85 countries/regions, and China was the most potential country. More than half of the top 10 most prolific organizations were located in the United States, and Harvard Medical School was the most important organization in this field. Nearly half of authors who make major contributions belonged to the United States organizations when publishing documents. *Frontiers in Immunology* was the most popular journal in this research area. Immunomodulation, gut microbiota, and cytokines represent the current research hotspots and frontiers in this field. Treg cell-based immunomodulatory approaches have shown immense potential in the treatment of neurological diseases. Modifying gut microbiota or regulating cytokines to boost the numbers and functions of Treg cells represents a promising therapeutic strategy for neurological diseases. Hence, we can conclude from these documents that future therapeutic strategies for neurological diseases should leverage the therapeutic potential of Treg cells, with an emphasis on modulating their activity to promote neuroprotection.

## Data availability statement

The original contributions presented in the study are included in the article/supplementary material, further inquiries can be directed to the corresponding author.

## Author contributions

QG: Conceptualization, Data curation, Writing—original draft, Writing—review and editing. XL: Conceptualization, Validation, Writing—review and editing. YL: Funding acquisition, Project administration, Validation, Writing—review and editing. JL: Methodology, Writing—review and editing. MP: Formal analysis, Writing—review and editing. JW: Visualization, Writing—review and editing. FY: Software, Writing—review and editing. YZ: Funding acquisition, Project administration, Supervision, Writing—review and editing.
